# Nonpharmacological Forms of Therapy to Reduce the Burden on Caregivers of Patients with Dementia—A Pilot Intervention Study

**DOI:** 10.3390/ijerph17249153

**Published:** 2020-12-08

**Authors:** Joanna Szczepańska-Gieracha, Lilianna Jaworska-Burzyńska, Krystyna Boroń-Krupińska, Joanna Kowalska

**Affiliations:** 1 Department of Physiotherapy, University School of Physical Education, 51-612 Wroclaw, Poland; joanna.szczepanska@awf.wroc.pl (J.S.-G.); lilianna.jaworska@op.pl (L.J.-B.); 2 Department of Physical Education and Sport, University School of Physical Education, 51-612 Wroclaw, Poland; krystyna.boron@awf.wroc.pl

**Keywords:** massage, relaxation, depression, caregiver burden, therapy, dementia

## Abstract

The aim of this pilot intervention study was to assess the effectiveness of selected forms of therapy (massage and relaxation) in reducing the perceived burden and improving the emotional status of caregivers of people with dementia and to determine which form of physical intervention is most effective. The study group was made up of 45 informal caregivers, who were divided into three subgroups (the massage group, relaxation group and control group). The Caregiver Burden Scale (CBS), Beck Depression Inventory (BDI), Berlin Social Support Scale (BSSS) and the Satisfaction with Life Scale (SWLS) were used. In the study group of caregivers, an average level of perceived burden, satisfactory life satisfaction and moderate severity of depressive symptoms were found. Massage led to a reduction in perceived burden and an improvement in mood and well-being of the examined group of caregivers. Group relaxation activities had no effect on the level of burden experienced by the caregivers, but significantly improved their mood. Both massage and relaxation were equally effective in improving the well-being of caregivers. Due to the lower cost of group activities, relaxation activities seem to be more effective and easier to organize, but further studies are necessary.

## 1. Introduction

The increased demand for psychogeriatric care is putting a strain on medical care systems in many countries, often requiring family and informal caregivers (family caregivers) to step in [1]. In Poland, as many as 92% of elderly people suffering from dementia live at home from the moment of diagnosis until death, and 44% of home caregivers provide assistance on their own without any outside support [2]. The need to take care of a patient is often associated with negative consequences for the caregivers themselves, referred to as the “caregiver burden” [3].

A good understanding of the disease and support from others can significantly improve the psychophysical condition of the caregiver [4]. Unfortunately, by focusing their attention on the patient (a relative with dementia), the caregiver often neglects their own needs. Day care or in-patient care facilities offer assistance to the patient, somewhat relieving the caregiver, but this assistance is not enough. Although caregivers’ social needs are high, very few centers pride themselves on providing long-term support groups for caregivers or having a permanent system of training for this group of individuals.

Given the many negative elements of caregiver burden, it is essential to provide a form of social support that meets the needs of caregivers in as many ways as possible, relatively fast and on a reasonably low budget. Only multidimensional actions that involve a “biopsychosocial” approach to the problem, including working with the body, psychotherapy, psychoeducation and social activation, achieve long-term results [5].

One of the methods used to reduce psychophysical tension is a relaxing massage [6]. Many studies have confirmed the positive impact of massage as part of treatment or support of various individuals (e.g., doctors, nurses, social workers, caregivers and psychotherapists) [7]. Relaxation techniques are also widely used with these types of problems (psychophysical tensions), particularly group relaxation exercises which have a strong impact due to their association with group work. Moreover, this type of intervention can be seen as a low-cost strategy [8].

Analysis of the literature on techniques to reduce burnout syndrome in different social groups revealed that no previous studies have connected all the elements of social support in relation to burden reduction in a particular group [7]. In most publications, support groups and exercises were conducted separately in different target groups [9]. Furthermore, the literature suggests that only a combination of support groups and body workouts (whether massage or relaxation exercises) provide the best results in reducing the perceived burden, which also applies to caregivers of people with dementia [7].

In this study, we assessed the effectiveness of selected forms of therapy (massage and relaxation) in reducing the perceived burden and improving the emotional status of caregivers of people with dementia. The aims of this study were to determine whether relaxing massages and group relaxation activities influence the level of perceived burden, intensity of depressive symptoms, level of perceived social support and life satisfaction in a group of caregivers of people with dementia, and to determine which form of intervention is most effective.

## 2. Materials and Methods

### 2.1. Participants and Procedure

This research presents the results of the second part of a project conducted in Wroclaw in 2015, “Alzheimer’s Cafe—a place for meeting, support and social integration”, co-funded by the Municipality of Wroclaw and run by the SIWY DYM, a Foundation for Senior Citizens Activation. The project lasted for a year and included a group of caregivers who participated in information training, support group sessions and individual consultations with a psychotherapist [3].

The study group comprised 58 informal caregivers, who applied to take part in the project to receive information support, after having read the information published in local media. This part of the project has been described in detail in a previous publication (in the *American Journal of Alzheimer’s Disease & Other Dementias*) [3].

After the training course, individuals who met the following inclusion criteria were invited to the next stage of the project: written consent to participate in the study and a declaration that they are the primary caregiver of the patient with dementia and receive no financial compensation. The following exclusion criteria were also applied: caring for a person with an illness other than dementia, previous experience caring for a patient with dementia, death of the patient during the research project, patient stayed in a hospital or another care facility during the caregiver’s involvement with the project, commencement of psychiatric (e.g., antidepressant) treatment of the caregiver during the research project, deterioration of the caregiver’s health preventing further participation in the project, and an inability to organize care for the dementia sufferer while the caregiver participated in activities related to the research project.

**Figure 1:**
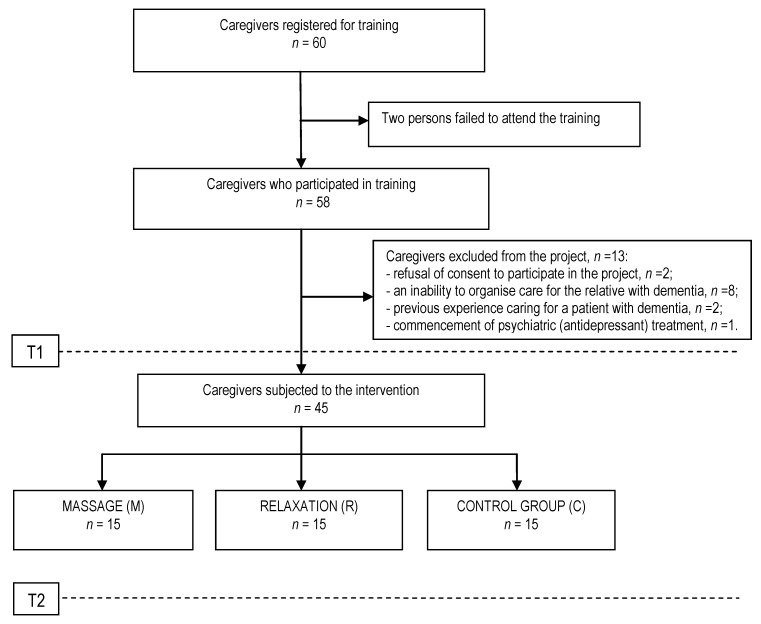
Study group selection.

A total of 45 caregivers qualified to participate in the project; they were randomly divided into three subgroups (Figure 1): group M, caregivers who participated in support groups and in 10 additional individual relaxation massage sessions; group R, caregivers who participated in support groups and in 10 additional group relaxation sessions; and group C, caregivers who only participated in support groups.

A relaxing massage was conducted in accordance with standard massage techniques and guidelines, performed by a trained massage therapist in a separate office. The subjects were instructed to undress freely and position themselves lying face down on a massage table. They underwent a total of 10 massage sessions performed five times per week, with each treatment session lasting 45 min. Standard relaxation massage techniques involving stroking and rubbing were applied, working the entire back with particular emphasis on the neck and around the spine. The main technique during the treatment was stroking, including longitudinal, transverse and circular movements around the buttocks and back; “figure eight”; shoulder and neck strokes; and kneading the muscles around the spine [10,11].

Relaxation training took place from Monday to Friday for two consecutive weeks, with each session lasting 45 min. The methods used included autogenic training according to Schultz, progressive muscle relaxation according to Jacobson, breathing exercises, the Trager approach, relaxation training according to Wintrebert, visualization exercises and elements of mindfulness practices [12,13].

Caregivers were informed about the aim and rules of the study and the possibility of withdrawing at any stage of the research.

Approval to conduct the study was obtained from the Bioethics Committee of the University School of Physical Education in Wroclaw (reference no. 18/2015). This study was conducted in accordance with the Helsinki Declaration.

### 2.2. Measure Tools

The following tests were used: the Caregiver Burden Scale (CBS), the Beck Depression Inventory (BDI), the Berlin Social Support Scale (BSSS), the Satisfaction with Life Scale (SWLS) and an information questionnaire.

The CBS consists of 22 items including 5 dimensions: general strain (GS), isolation (I), disappointment (D), emotional involvement (EI), and environmental burden (EB). The CBS has satisfactory psychometric properties and is used to measure the burden of caring for dementia patients on caregivers [14]. The Polish adaptation was developed by Jaracz and Grabowska-Fudala. Higher scores indicate a higher burden on the respondent. The following scores of burden were adopted: low level (1.00–1.99 points), medium level (2.00–2.99 points), and high level (3.00–4.00 points) [15].

The BDI contains 21 items that relate to the most significant symptoms of depression. The first 13 questions focus on cognitive-affective aspects, while the remaining questions relate to somatic symptoms. The Polish version of the BDI has very good psychometric properties: Cronbach’s α was 0.95 for a clinical trial and 0.93 for a control group (similar to the original version). Scores from 0 to 11 points indicate no depressive disorders, with higher scores indicating more depressive symptoms [16].

The BSSS measures the cognitive and behavioural dimensions of social support and consists of 4 independent scales of social support: perceived availability of support (BSSS I), need for support (BSSS II), seeking support (BSSS III) and currently receiving support (BSSS IV). The Polish adaptation was developed by Łuszczyńska et al. and has satisfactory psychometric properties (Cronbach’s α ranges from 0.71 to 0.90) [17].

The SWLS measures the subject’s subjective sense of life satisfaction. The higher the score, the more satisfied with life the respondent is. The following raw results of the Polish standards were used: 5–17 points, low satisfaction; 18–23 points, average satisfaction; 24–35 points, high satisfaction. The psychometric properties of the Polish version are satisfactory and similar to the original [18,19].

The above tests were conducted at two time points, the first of which (T1) occurred before the commencement of group support. The intervention involved a 6-month group support cycle (one 3-h meeting per month), with an additional massage or relaxation procedure applied in the final month depending on the subgroup. After the completion of all therapeutic activities, the final tests (T2) were carried out.

### 2.3. Data Analysis

The study group was characterised using descriptive statistics, including mean, standard deviation, minimum and maximum values and, in the case of qualitative variables, numbers and percentages. The Kolmogorov–Smirnov test was used to check for normal distribution of the data. The data were normally distributed; therefore, a Student’s *t*-test was applied for both dependent and independent samples. Comparisons of the three groups (M vs. R vs. C) were carried out using a one-way ANOVA with Scheffe’s post hoc test. Changes in the distribution of answers to individual questions (variables in the order scale) were analysed using Wilcoxon’s ranked differences test. Pearson’s linear correlation coefficient was used to describe correlations between continuous variables. The significance threshold was set at *p* < 0.05. The calculations were performed using STATISTICA 12.

## 3. Results

The study group consisted of 45 caregivers, including 40 women and 5 men. Detailed characteristics of this group and the dementia sufferers they cared for are presented in Table 1.

There were no statistically significant differences at the first measurement point (T1) between the massage (M), relaxation (R) and control (C) subgroups in the age distribution of caregivers, age of the patients, degree of kinship, duration of the patients’ illness, or in the frequency and intensity of care. Statistically, the shortest care time was recorded in group C (*p* = 0.011). There were no statistically significant differences in CBS, BDI, SWLS and BSSS between the groups at the T1 measurement point. Significant between-group differences were observed only in the BSSS-2 (need for support) and BSSS-3 (seeking support) subscales. The lowest mean values for these subscales were recorded in group R.

After interventions (T2), the mean level of burden in group R was decreased compared to the initial test values (T1); however the difference observed was not statistically significant. Statistically significant changes in the CBS and GS subscales (a decrease in burden level) were observed in group M, whereas an increase in burden level was observed in group C. This change was not statistically significant. The ANOVA revealed statistically significant differences between the T2 and T1 time points in the studied groups (M vs. R vs. C) for the total CBS score and the GS subscale. The results are presented in Table 2. Scheffe’s post hoc test for the total CBS score and GS variables revealed that the greatest changes occurred in group C (Table 3).

At T2, the mean total BDI score and the mean scores for emotional and somatic subscales were significantly decreased in the M and R groups but significantly increased in group C. There were also significant intergroup differences in the total BDI score and in both subscales, with significant differences in group C compared to the other groups. Mean changes in the M and R groups did not differ significantly (Table 2 and Table 4).

Additional qualitative analysis was performed for the BDI scores in the M and R groups to identify aspects of the questionnaires that showed significant changes between T1 and T2 (Wilcoxon signed-rank test). A statistically significant change in the BDI scale was found in group M for the B, D, J and T dimensions, while in group R, the K, M and T dimensions were significantly different (Table 5 and Table 6). In group R, the level of anxiety of caregivers decreased as a result of participation in the intervention. Moreover, they found it easier to make decisions and felt less concerned about their own health. In group M, fears about the future were decreased, everyday life had become less burdensome, and tearfulness and fears about their own health were also decreased.

Although there were slight changes in the mean BSSS results in the final test compared to the initial test in groups M, R and C, this result was not statistically significant. There were no significant intergroup differences in the studied parameters. Similar results were also observed for SWLS.

Correlation analysis for the whole group of caregivers (*n* = 45) indicated a statistically significant (although moderate) positive relationship between the CBS results and the age of caregivers. The remaining correlations between the CBS and BDI results with selected characteristics of care work undertaken by caregivers were weak and not statistically significant (Table 7).

Male caregivers were found to be in a significantly worse mood at the beginning of therapy compared to women. In addition, caregivers who received no assistance showed significantly higher levels of burden and a worse mood compared to those who received help (Table 8). These results, although statistically significant, need to be verified on a larger sample due to the small number of unassisted cases and small number of males.

## 4. Discussion

The results of our study reveal that the care of people with psychogeriatric disorders in Poland predominantly rests on the family. The longer life expectancy of females is one of the reasons behind the prevalence of females as caregivers, as evidenced by previous research [15,20,21]. Institutional assistance is provided to only 8% of caregivers in Poland, mainly in large cities, compared to over 50% in Scandinavian countries [22]. This may be due not only to a lack of available assistance, but also to a strong sense of responsibility for the care of older family members.

In the present research project, intergroup variations in caregiver burden and its components did not differ significantly between the three groups (the massage, relaxation and control groups). After a series of massage sessions, a decrease in all burden components was observed. Statistically, the total score improved significantly (*p* = 0.007), while the GS experienced by the caregiver significantly decreased (*p* = 0.001). Similar findings were obtained for caregivers of oncology patients [23]. In the relaxation group, the burden level also decreased; however, the results did not reach statistical significance. In 2013, Pitteri et al. conducted a study on a group of 50 caregivers of people with Alzheimer’s disease. The results showed that combining relaxation techniques with a psychoeducational intervention has a positive effect on the perceived burden in the study group [24]. Similarly, burden reduction was achieved in a study by Ali and Bokharey in 2015, who investigated the combination of relaxation techniques with psychoeducation. However, the researchers used a different caregiver burden measurement scale, and the project itself took longer to complete, as much as 3600 h [25]. A significant decrease in caregiver burden was observed in studies on the impact of low intensity physical activity on caregivers of people suffering from dementia [26]. In this case, the group nature of these exercises may be of significant importance.

In our study, both massage and relaxation contributed to a decreased perception of burden in caregivers, with the most noticeable changes occurring in the massage group, particularly in terms of the decrease in GS (general strain). Interestingly, during this time, all examined parameters related to the feeling of burden increased in the control group. A statistically significant difference was recorded in the subscale associated with a feeling of disappointment (D). This suggests that information support alone in the form of a one-off training session and subsequent participation in a support group (held once a month) is not sufficient to significantly reduce the level of caregiver fatigue. Studies by other authors confirm this thesis [12,27,28,29]. The use of information support alone did not improve the sense of SI (social isolation), which has also been confirmed by other authors [30]. Furthermore, it should be stressed that in this research, all caregivers who were qualified and participated in the second stage of the study (*n* = 45) expressed a need for additional support in the form of massage or relaxation activities (apart from meetings with a psychotherapist). The control group, which did not receive additional support in the form of body work, was chosen by a randomized draw in line with the assumptions of the project. This may have contributed to the increased sense of disappointment (D) observed in group C.

Another parameter related to the mental state of caregivers examined in the current study was the intensity of mood disorders. The mean total BDI score for the whole group was 13.6 points, which indicates depression and signals a need for psychiatric consultation to verify the diagnosis. This result provides only informative value in terms of the mental condition of the whole group, as both people with severe symptoms of depression and those with a good mental status were included. In a study by Gustaw et al., caring for a suffering family member provided a sense of satisfaction, a sense of duty towards loved ones, strength and control over oneself and the situation, as well as a sense of self-esteem [21]. In research by Grochowska, as many as 39% of caregivers felt satisfaction with the care they had provided at least sometimes, with 36% reporting often and 20% always [31]. However, the above does not change the fact that family caregivers need support in the difficult task of providing care to an ill family member. This was also confirmed by correlation analysis, which showed a significant relationship between the level of depression and the amount of support the caregiver receives. Lack of support and increasing symptoms of depression determined higher levels of caregiver burden in the study group and vice versa. A high level of support was found to guarantee a good mental state of the caregiver [3].

When the three groups were compared, the mood and well-being of caregivers did not differ significantly, both in terms of the total score and when they were divided into emotional and somatic subscales. In the group that received the massage intervention, the total BDI score significantly decreased, as well as the emotional and somatic subscales. Interestingly, after 10 massage sessions, the respondents declared that they were less concerned about the future, enjoyed the present more often, were less anxious and less worried about their own health. Similar results were obtained in other groups of caregivers following massage [23,32,33,34]. Additionally, a statistically significant decrease in the level of depression was achieved as a result of the relaxation classes, which was evident in the total score and in the emotional and somatic subscales. After 10 relaxation sessions, the subjects claimed that they were less nervous on a daily basis, made decisions more easily and did not worry as much about their own health. Studies by other authors demonstrated a decrease in depression among caregivers of oncology patients following group strength training [35].

Unfortunately, during the 6-month observation period, the level of depression in the control group increased significantly, both in the total score and in the emotional subscale. Similar results have also been reported by other researchers [36,37,38]. Longston et al. argued that the best type of support for caregivers of people suffering from dementia is a support group consisting of participants in the same situation who come together to help and support each other with coping, improving their own psychological functioning and increasing their effectiveness [39]. The research conducted in this project demonstrates that it is not only the quality but also the frequency of the measures undertaken that are important. It seems that meetings held once a month did not serve their purpose. However, this was a pilot project, and significant changes need to be introduced based on the findings, such as an increased frequency of meetings. Moreover, organisational support for caregivers needs to be provided, ensuring that care of the dementia patient is established during the regular therapeutic activities dedicated to the caregivers. Both the results of our research and the reports of other authors cited above demonstrate the importance of activities aimed at supporting caregivers in the biopsychosocial sphere, including informational support, emotional support, social activation (through group activities) and body work.

In this study, the level of perceived support was 97.6 points, which indicates a relatively high level of support experienced by the respondents. The level of perceived life satisfaction was also satisfactory (20.3 points). No significant changes in the area of life satisfaction were observed in any of the subgroups during the project. We learned from other reports that providing a massage intervention to caregivers improved their quality of life, mainly their sleep [40]. Reports from Basińska et al. show that the higher the level of support, the lower the level of fatigue of the caregiver, especially physical fatigue [41]. The literature shows that caregivers of Alzheimer’s patients have a particular need for psychological support. Almost all respondents identified a need for psychological and psychotherapeutic interventions. Caregivers are seriously affected by a lack of help and support from their family, society and the institutions that were established to support them [21]. The results of this research differ significantly from those presented above due to the fact that the vast majority of the studied group (84%) were able to rely on help with care, which ultimately meant that they were able to take part in the project.

In studies of a social nature, many variables influence the mental state of people under observation. Throughout the whole research project, its participants functioned in their natural environment and, apart from the organized therapeutic activities, they had more or less opportunities to establish social contacts, which is very important in assessing the burden and level of social support. In further research, this variable should be analyzed more broadly.

In summary, the findings presented herein support the 2017 recommendations of the World Health Organization (WHO), which launched a global action plan on dementia care that was approved by the WHO and ratified by 194 Member States. The plan places particular emphasis on training in terms of the skills required to care for others, as well as on social support for the caregivers [42]. This research has shown that complementing informational and emotional support with additional therapeutic factors (massage and relaxation) significantly increases the effectiveness of such activities by reducing psychophysical tension, reflected in better mental state parameters (enhanced well-being and mood), bringing relief to caregivers at the somatic level and increasing future chances of care being provided by the caregivers. Home care has many advantages over institutional care, so it is worth identifying ways and means to support caregivers in this challenging task.

### Limitations

This study was screening in nature, thus the findings obtained are not equal to a medical diagnosis. One of the limitations of this project was the relatively small group of respondents, with poor representation of male caregivers and those individuals who did not receive assistance. Another serious problem was the lack of objective (research-based) knowledge about the psychophysical condition of the dementia sufferers, which is of great importance in terms of the amount of effort that needs to be put into the care of the patient. Nonetheless, caregivers do not usually compare their patients to other people with similar problems or subjectively experience the difficulties involved in caring for others because they are not familiar with their situation. Future research should be expanded to include the somatic condition of the caregivers and the ways in which they spend their free time. It is also worth extending the panel of psychometric tests to include tools to assess the level of caregivers’ anxiety and stress, as well as to determine the best methods to deal with stress. It would also be valuable to measure stress biomarkers before and after the therapeutic intervention. These studies should be continued, ideally taking into account the above remarks, on a larger number of respondents to identify measures that will most effectively support caregivers in their future care of a suffering family member. Furthermore, we have no knowledge of how long the effects of the nonpharmacological forms of therapy (massage and relaxation) last after being provided. Moreover, for the purposes of this scientific project, the selection of the groups was random, thus. the intervention may not have been the most suitable for a given person. It is possible that the caregiver themselves should be the one to choose the form of support from several proposals that are available, adjusting their choice to their own abilities and preferences.

## 5. Conclusions

1. In the group of caregivers, average levels of perceived burden, satisfactory life satisfaction and moderate severity of depressive symptoms were found.
2. Relaxing massages led to a reduction in perceived burden and an improvement in mood and well-being of the examined group of caregivers compared to the control group.
3. Group relaxation activities had no effect on the level of burden experienced by the caregivers, but significantly improved their mood and well-being compared to the control group.
4. In the control group, a deterioration of mental state and perceived burden was observed during the project.
5. There were no differences between relaxing massages and group relaxation activities in terms of reducing the perceived burden and improving the psychophysical condition of caregivers. Both forms were equally effective. Due to the lower cost of group activities, this form of support for caregivers (group relaxation activities) seems to be more effective and easier to organize.

## Figures and Tables

**Table 1:** Baseline characteristics of the study group (*n* = 45) and patients with dementia.

Caregiver Characteristics	*n* (%)
Sex	
Female	40 (89)
Male	5 (11)
Age	
Mean (SD)	56.8 (11.5)
CBS	
Mean (SD)	59.6 (11.9)
BDI	
Mean (SD)	13.6 (6.3)
BSSS	
Mean (SD)	97.6 (15.6)
SWLS	
Mean (SD)	20.3 (6.1)
Duration of care (years):	
1–2	8 (18)
3–4	13 (29)
5–6	12 (27)
7–10	8 (18)
>10	3 (7)
No data	1 (2)
Time frame of care:	
Ongoing	24 (53)
Sometimes	19 (42)
In special situations	2 (4)
Who assists the caregivers:	
Family	31 (69)
Nobody	7 (16)
Professionals	4 (9)
Family and Professionals	1 (2)
Others	2 (4)
How often caregivers receive support from others:	
Often	30 (67)
Sometimes	8 (18)
Never	7 (16)
**Characteristics of patients**	
Age	
Mean (SD)	81.6 (7.5)
Relationship of patient to caregiver:	
Mother	21 (46)
Father	8 (18)
Husband/wife	8 (18)
In-law	3 (7)
Grandfather/grandmother	4 (9)
Others	1 (2)
Duration of illness (years)	
Mean (SD)	5.8 (3.7)

CBS, Caregiver Burden Scale; BDI, Beck Depression Inventory; BSSS, Berlin Social.

**Table 2:** Differences in the studied parameters (CBS and BDI) over time (Student’s *t*-test for dependent groups) and comparisons between the three groups (ANOVA).

Variables		Group	T1	T2	Difference (T2-T1) Mean (SD)	Student’s *t*-Test		ANOVA	
			Mean (SD)	Mean (SD)		*t*	*p*	F	*p*
CBS	Total score	M	57.5 (10.8)	52.2 (13.1)	−5.3 (6.4)	3.2	0.007 *	6.6	0.003 *
		R	59.9 (12.3)	57.1 (11.8)	−2.8 (7.4)	−1.5	0.163		
		C	59.5 (13.3)	63.3 (12.5)	3.8 (5.5)	1.4	0.193		
	GS	M	23.0 (5.4)	19.9 (5.1)	−3.1 (2.8)	4.3	0.001 *	11.9	0.0001 *
		R	22.7 (5.7)	22.1 (3.8)	−0.6 (3.4)	−0.7	0.505		
		C	22.8 (5.1)	25.2 (4.2)	2.4 (2.3)	1.07	0.301		
	SI	M	7.1 (2.6)	6.7 (2.1)	−0.4 (2.1)	0.7	0.472	1.9	0.164
		R	8.3 (1.9)	7.3 (2.3)	−1.0 (2.04)	−1.9	0.078		
		C	8.2 (2.3)	8.7 (2.0)	0.5 (1.8)	1.3	0.210		
	D	M	12.7 (4.1)	11.9 (3.3)	−0.8 (3.9)	0.7	0.476	0.04	0.956
		R	13.5 (3.4)	12.8 (3.4)	−0.7 (2.0)	1.5	0.142		
		C	14.4 (2.9)	13.6 (3.7)	−0.8 (2.1)	2.2	0.042 *		
	EI	M	7.3 (1.9)	6.9 (2.5)	−0.4 (1.9)	0.9	0.354	0.3	0.755
		R	7.6 (1.8)	7.3 (2.2)	−0.3 (2.1)	1.2	0.238		
		C	7.7 (1.6)	7.3 (1.8)	−0.4 (0.6)	1.8	0.085		
	EB	M	7.3 (1.9)	6.8 (2.0)	−0.5 (2.2)	0.9	0.357	3.2	0.053
		R	7.1 (2.3)	6.8 (2.7)	−0.3 (1.5)	−0.8	0.417		
		C	6.9 (2.9)	8.5 (2.9)	1.6 (3.3)	0.4	0.705		
BDI	Total score	M	13.9 (4.2)	7.9 (5.2)	−6.0 (5.5)	4.2	0.001 *	10.0	0.0003 *
		R	13.1 (7.4)	8.4 (5.2)	−4.7 (6.9)	−2.6	0.020 *		
		C	12.3 (7.2)	15.8 (7.0)	3.5 (4.6)	2.7	0.022 *		
	Emotional subscale	M	8.1 (3.6)	4.6 (3.3)	−3.5 (3.7)	3.7	0.002 *	7.6	0.0017 *
		R	7.5 (4.4)	4.7 (3.3)	−2.8 (4.4)	−2.5	0.025 *		
		C	7.4 (4.8)	9.0 (4.7)	1.6 (2.2)	2.5	0.029 *		
	Somatic subscale	M	5.8 (2.2)	3.3 (2.4)	−2.5 (3.1)	3.1	0.008 *	7.6	0.002 *
		R	5.5 (3.5)	3.7 (2.6)	−1.8 (3.1)	−2.2	0.041 *		
		C	4.8 (3.6)	6.7 (2.7)	1.9 (3.1)	2.2	0.053		

CBS, Caregiver Burden Scale; GS, general strain; SI, social isolation; D, disappointment; EI, emotional involvement; EB, environmental burden; BDI, Beck Depression Inventory; M, massage group; R, relaxation group, C, control group; T1, initial results before therapy; T2, final results after therapy; * statistically significant (*p* < 0.05).

**Table 3:** Scheffe’s post hoc test for the CBS variables (total score and GS) between the massage (M), relaxation (R) and control (C) groups.

Group	CBS Total Score			GS		
	M {1}	R {2}	C {3}	M {1}	R {2}	C {3}
M {1}		0.5911	0.0042 *		0.0726	0.0001 *
R {2}	0.5911		0.0458 *	0.0726		0.0387
C {3}	0.0042 *	0.0458 *		0.0001 *	0.0387 *	

CBS, Caregiver Burden Scale; GS, general strain; M, massage group; R, relaxation group; C, control group; * statistically significant (*p* < 0.05).

**Table 4:** Scheffe’s post-hoc test for the BDI variables between the massage (M), relaxation (R) and control (C) groups.

Group	BDI-Total Score			Emotional Subscale			Somatic Subscale		
	M {1}	R {2}	C {3}	M {1}	R {2}	C {3}	M {1}	R {2}	C {3}
M {1}		0.823	0.001 *		0.882	0.003 *		0.840	0.003 *
R {2}	0.823		0.004 *	0.882		0.012 *	0.840		0.013 *
C {3}	0.001 *	0.004 *		0.003 *	0.012 *		0.003 *	0.013 *	

BDI, Beck Depression Inventory; M, massage group; R, relaxation group, C, control group; * statistically significant (*p* < 0.05).

**Table 5:** Qualitative differences in particular dimensions from Beck’s Depression Scale (BDI) for the massage group (M).

Dimensions	Mean		Difference (T2-T1)	Wilcoxon Test		
	T1	T2		T	Z	*p*
A	0.80	0.47	−0.33	10	1.48	0.139
B	1.00	0.53	−0.47	0	2.37	0.018 *
C	0.47	0.47	0.00	14	0.00	1.000
D	0.73	0.33	−0.40	0	2.20	0.028 *
E	0.73	0.47	−0.27	14	1.07	0.286
F	0.27	0.13	−0.13	0	1.34	0.180
G	0.47	0.20	−0.27	0	1.83	0.068
H	0.20	0.00	−0.20	0	1.60	0.109
I	0.00	0.00	0.00			
J	0.80	0.20	−0.60	0	2.20	0.028 *
K	1.00	0.60	−0.40	14	1.38	0.169
L	0.87	0.47	−0.40	4	1.77	0.076
M	0.80	0.73	−0.07	12	0.34	0.735
N	0.60	0.53	−0.07	2	0.53	0.593
O	0.40	0.33	−0.07	12	0.34	0.735
P	0.53	0.47	−0.07	6	0.40	0.686
Q	0.73	0.60	−0.13	7	0.73	0.463
R	0.33	0.13	−0.20	0	1.34	0.180
S	1.00	0.27	−0.73	4	1.69	0.091
T	1.20	0.33	−0.87	3	2.37	0.018 *
U	1.00	0.67	−0.33	7	1.18	0.237

T1, initial results before therapy; T2, final results after therapy; * statistically significant (*p* < 0.05).

**Table 6:** Qualitative differences in particular dimensions from Beck’s Depression Scale (BDI) for the relaxation group (R).

Dimensions	Mean		Difference (T2-T1)	Wilcoxon Test		
	T1	T2		T	Z	*p*
A	0.67	0.47	−0.20	0	1.34	0.180
B	1.00	0.87	−0.13	0	1.34	0.180
C	0.40	0.07	−0.33	0	1.83	0.068
D	0.79	0.53	−0.20	5	0.81	0.418
E	0.73	0.43	−0.29	0	1.83	0.068
F	0.40	0.00	−0.40	0	1.60	0.109
G	0.40	0.60	0.20	6	0.94	0.345
H	0.40	0.13	−0.27	0	1.83	0.068
I	0.07	0.07	0.00			
J	0.27	0.13	−0.13	2	0.80	0.423
K	0.80	0.47	−0.33	0	2.02	0.043 *
L	0.60	0.33	−0.27	3	1.35	0.178
M	1.07	0.60	−0.47	0	2.20	0.028 *
N	0.67	0.33	−0.33	2	1.48	0.138
O	0.33	0.53	0.20	3	1.21	0.225
P	1.20	0.73	−0.47	3	1.86	0.063
Q	0.80	0.60	−0.20	3	1.21	0.225
R	0.53	0.20	−0.33	0	1.83	0.068
S	0.27	0.40	0.13	3	0.91	0.361
T	1.00	0.47	−0.53	4	2.03	0.042 *
U	0.73	0.47	−0.27	0	1.60	0.109

T1, initial results before therapy; T2, final results after therapy; * statistically significant (*p* < 0.05).

**Table 7:** Pearson’s linear correlation coefficient (r) of selected characteristics of care undertaken with the results of CBS and BDI scales at the T1.

Characteristic	CBS (T1)	BDI (T1)
Caregiver age	0.53 *	0.04
Patient age	−0.03	0.05
Duration of patient’s illness	0.04	−0.07
Duration of care	0.04	−0.19

CBS, Caregiver Burden Scale; BDI, Beck Depression Inventory; T1, initial results before therapy; * statistically significant.

**Table 8:** Comparison of the CBS and BDI results according to gender and assistance received (Student’s *t*-test for independent groups).

Characteristic	CBS (T1)		Student’s *t*-Test		BDI (T1)		Student’s *t*-Test	
	Mean	SD	t	p	Mean	SD	t	p
Sex								
Female (*n* = 40)	58.9	11.6	−1.04	0.1516	14.2	6.2	1.78	0.0411 *
Male (*n* = 5)	64.8	13.7			9.0	5.9		
Support received								
Yes (*n* = 38)	57.6	11.1	−2.83	0.0035 *	12.8	6.0	−2.12	0.0198 *
No (*n* = 7)	70.4	10.4			18.1	6.4		

CBS, Caregiver Burden Scale; BDI, Back Depression Inventory; T1, initial results before therapy; * statistically significant (*p* < 0.05).
